# Inhibition of Klf10 Attenuates Oxidative Stress-Induced Senescence of Chondrocytes via Modulating Mitophagy

**DOI:** 10.3390/molecules28030924

**Published:** 2023-01-17

**Authors:** Jie Shang, Nan Lin, Rong Peng, Ning Jiang, Biao Wu, Baizhou Xing, Shiyuan Lin, Xianghe Xu, Huading Lu

**Affiliations:** 1 Department of Orthopedics, The Fifth Affiliated Hospital of Sun Yat-sen University, Zhuhai 519000, China; 2 Guangdong Provincial Key Laboratory of Biomedical Imaging, The Fifth Affiliated Hospital of Sun Yat-sen University, Zhuhai 519000, China; 3 First Department of Orthopedics, Zhongshan City People’s Hospital, Zhongshan 528400, China

**Keywords:** osteoarthritis, Klf10, senescence, mitochondria, autophagy

## Abstract

Osteoarthritis (OA) is the most prevalent degenerative joint disease in the elderly. Accumulation of evidence has suggested that chondrocyte senescence plays a significant role in OA development. Here, we show that Krüppel-like factor 10 (Klf10), also named TGFβ inducible early gene-1 (TIEG1), is involved in the pathology of chondrocyte senescence. Knocking down the Klf10 in chondrocytes attenuated the tert-butyl hydroperoxide (TBHP)-induced senescence, inhibited generation of reactive oxygen species (ROS), and maintained mitochondrial homeostasis by activating mitophagy. These findings suggested that knocking down Klf10 inhibited senescence-related changes in chondrocytes and improved cartilage homeostasis, indicating that Klf10 may be a therapeutic target for protecting cartilage against OA.

## 1. Introduction

Osteoarthritis (OA) is a common degenerative joint disease that often occurs in patients over 65 years old and affects about 16% of the global population [1]. OA is characterized by joint pain, dysfunction, and deformities. It most commonly affects weight-bearing joints such as the knees and hips, and is one of the leading causes of lower limb disability in the elderly [2]. However, the underlying mechanisms of OA pathogenesis remain unknown.

Cellular senescence refers to the state of irreversible cell-cycle arrest in which cells become resistant to growth-promoting stimuli [3]. In addition to cell-cycle arrest, senescent cells exhibit certain common features, including increased activity of acidic lysosomal β-galactosidase [4], activation of p21 (CIP1) and p16(INK4a) [4,5], and enhanced secretion of senescence-associated secretory phenotype (SASP) including various proinflammatory cytokines, chemokines, and proteases [6,7]. Experimental data from several studies suggests that chondrocyte senescence plays an important role in OA [8,9,10]. Gao et al. reported that the expression of senescence-associated beta-galactosidase (SA-β-Gal) in the articular cartilage of knee OA was positively correlated with lesions in OA cartilage [11]. An in vivo study revealed that selective removal of senescent chondrocytes reduced the expression of inflammatory markers while increasing the expression of extracellular matrix proteins in chondrocytes [12]. Multiple factors can account for cellular senescence. 41Oxidative stress, caused by excessive intracellular reactive oxygen species (ROS), is regarded as an important factor leading to cellular senescence [13,14].

Autophagy is a conserved system that relies on the lysosomal pathway to degrade misfolded proteins or damaged organelles, thereby fulfilling cellular renovation and maintaining cellular homeostasis under stress conditions [15]. Increasing evidence has demonstrated a close link between defective autophagy and cellular senescence in multiple tissues [16,17,18]. Mitochondria are specialized metabolic organelles required for oxidative respiration and energy production in eukaryotic cells [19]. In senescent cells, mitochondria underwent various changes pertaining to dynamics, structure, and function [20,21] and exhibited multiple abnormities including increased ROS generation, decreased mitochondrial membrane potential (MMP), and reduced adenosine triphosphate (ATP) production [22,23]. Mitophagy, as selective autophagy, is an essential mechanism to eliminate dysfunctional mitochondria. However, weakened mitophagy within senescent cells led to abnormal accumulation of dysfunctional mitochondria and elevated levels of intracellular ROS [24,25].

Krüppel-like factor 10 (Klf10), also known as TGFβ inducible early gene-1 (TIEG1), is a transcriptional regulator of the Krüppel-like family and was first discovered in normal human fetal osteoblasts [26]. Klf10 has been linked to multiple cellular biological processes, including cell proliferation, apoptosis, and differentiation [27,28,29]. Zhou et al. reported that inhibiting KLF10 repressed glucose-induced elevated ROS generation by transcriptionally regulating heme oxygenase (Ho-1) [30]. Furthermore, Klf10 downregulation was able to alleviate radiation-induced apoptosis and promote damaged DNA repair and autophagy [31]. However, few studies have focused on the role of Klf10 in the development of OA. Our current study suggested that Klf10 was upregulated in senescent chondrocytes. Downregulation of Klf10 in primary mouse chondrocytes attenuated cellular senescence, inhibited ROS generation, and maintained mitochondrial homeostasis through the activation of mitophagy. 

## 2. Results

### 2.1. Klf10 Was Overexpressed in Senescent Chondrocytes and OA Cartilage

**Figure 1:**
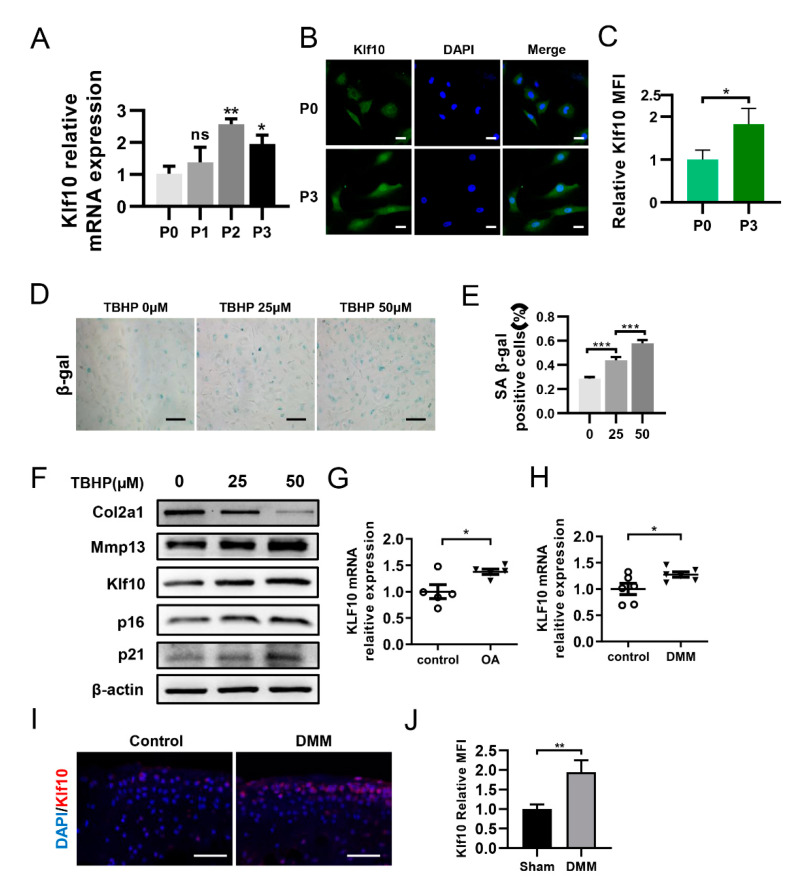
Klf10 was upregulated in senescent chondrocytes and OA cartilage. (**A**) Relative Klf10 mRNA expression levels of murine chondrocytes from P0–P3 were detected using RT-qPCR (*n* = 3). (**B**,**C**) Klf10 protein expression in P0 and P3 murine chondrocytes was detected by immunofluorescence (*n* = 3). Scale bar: 20 μm. (**D**,**E**) The murine chondrocytes were treated with 0, 25, or 50 μM TBHP for 48 h, and SA-β-gal staining was applied to detect senescent chondrocytes (*n* = 3). Scale bar: 200 μm. (**F**) The murine chondrocytes were treated with 0, 25, or 50 μM TBHP for 48 h. The expressions of Col2a1, Mmp13, p16, p21 and Klf10 protein were detected by Western blotting. (**G**) Relative Klf10 mRNA expression of human OA cartilage was detected using RT-qPCR (*n* = 5 patients per group). (**H**) Relative Klf10 mRNA expression of DMM mouse OA cartilage was detected using RT-qPCR (*n* = 6 mice per group). (**I**,**J**) Representative images of IF showing the upregulated expression of Klf10 in the DMM mouse OA model. Scale bar: 50 μm. Data are presented as the mean  ±  SD. Student’s *t* test was used for comparisons between two groups and one-way ANOVA with Tukey’s post hoc test was used for comparisons among multiple groups. * *p* < 0.05, ** *p* < 0.01, *** *p* < 0.001.

RT-qPCR was employed to determine the transcriptional level of Klf10 in primary chondrocytes at passages 0 to 3. The results showed that as the passage number increased, the mRNA expression of Klf10 was elevated (Figure 1A). Furthermore, immunofluorescence results revealed that Klf10 expression was significantly higher at passage 3 than at passage 0 (Figure 1B,C). Then, different doses of TBHP (0, 25, and 50 μM) were applied to induce chondrocyte senescence. SA-β-gal staining showed that the rate of SA-β-gal positive cells was highest at 50 µM TBHP (Figure 1D,E). Similarly, the expressions of Klf10 and senescent markers p16 and p21 were highest at 50 µM TBHP (Figure 1F). Furthermore, Col2a1 expression was reduced while Mmp13, an indicator of ECM degradation, was upregulated when the concentration of TBHP increased. Hence, a concentration of 50 μM TBHP was selected as optimal for further experiments. Furthermore, RT-qPCR revealed that the human OA cartilage and DMM mouse OA cartilage exhibited an upregulated Klf10 mRNA expression (Figure 1G,H). Immunofluorescence (IF) revealed increased Klf10 protein expression in DMM mouse OA cartilage (Figure 1I,J). All these results suggest that Klf10 expression was upregulated in senescent chondrocytes and OA cartilage.

### 2.2. Knocking down Klf10 Attenuated TBHP-Induced Senescence

**Figure 2:**
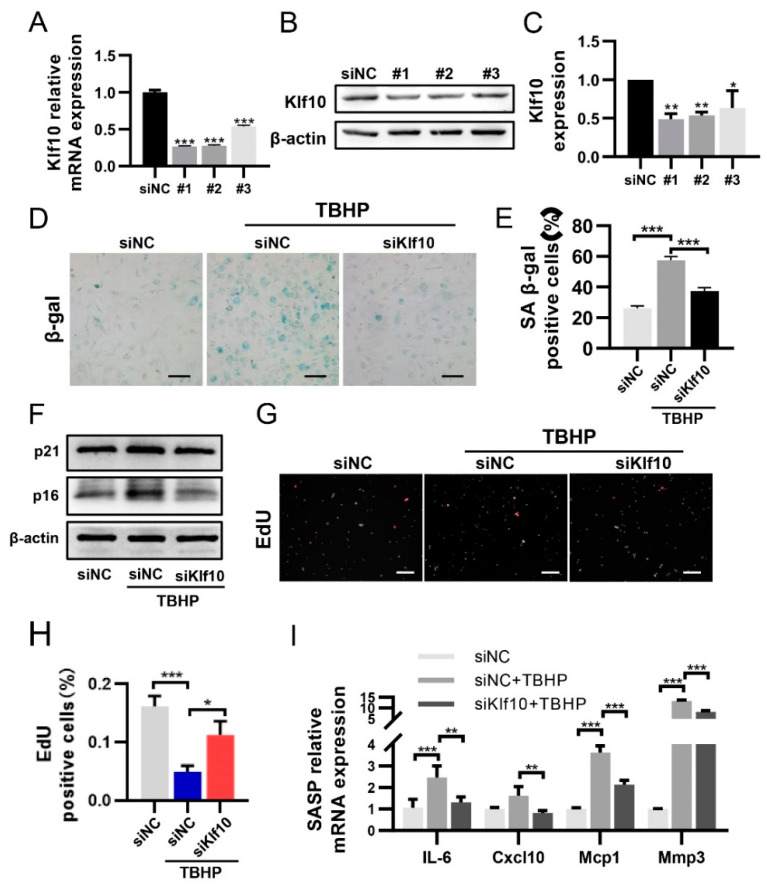
Knocking down Klf10 attenuated TBHP-induced senescence. (**A**) The murine chondrocytes were transfected with Klf10 siRNA #1, #2, #3, or negative control siRNA for 48 h. The relative Klf10 mRNA expression was detected using RT-qPCR (*n* = 3). (**B**,**C**) The murine chondrocytes were transfected with Klf10 siRNA #1, #2, #3, or negative control siRNA for 72 h. The relative Klf10 protein expression was detected by Western blotting (*n* = 3). The murine chondrocytes were treated with TBHP with or without transfection with siKlf10. (**D**,**E**) SA-β-gal staining was applied to detect the senescent chondrocytes in each group (*n* = 3). Scale bar: 200 μm. (**F**) Protein expression of p16 and p21 was detected by Western blotting. (**G**,**H**) Representative images of EdU-positive chondrocytes in each group (*n* = 3). Scale bar: 200 μm. (**I**) Relative mRNA expressions of Il-6, Cxcl10, Mcp1, and Mmp3 were analyzed using RT-qPCR (*n* = 3). Data are presented as the mean  ±  SD. One-way ANOVA with Tukey’s post hoc test was used for comparisons among multiple groups. * *p* < 0.05, ** *p* < 0.01, *** *p* < 0.001.

To investigate whether Klf10 played a functional role in chondrocyte senescence, Klf10-siRNA was applied in order to knock down Klf10. Three different Klf10 siRNA sequences were designed. As shown in Figure 2A–C, siKlf10 #1 displayed the best knockdown efficiency at both mRNA and protein levels in murine chondrocytes and was therefore chosen for the subsequent experiments. The SA-β-gal staining showed that knocking down Klf10 attenuated SA-β-gal activity induced by TBHP (Figure 2D,E). Likewise, expression of p16 and p21 was found to increase in the TBHP-treated group and decreased significantly when Klf10 was knocked down (Figure 2F). Proliferation arrest is an important feature of senescent cells, and the proliferation of chondrocytes was detected using the EdU assay. As displayed in Figure 2G,H, EdU-positive cells were found to decrease in the TBHP-treatment group, while knocking down Klf10 reversed this trend. Furthermore, mRNA expressions of SASP, including IL-6, Cxcl10, Mcp1, and Mmp3 were elevated in the TBHP group but were reversed when Klf10 was knocked down (Figure 2I). Taken together, these results suggested that knocking down Klf10 attenuated chondrocyte senescence and inhibit the secretion of SASP.

### 2.3. Downregulating Klf10 Attenuated Senescence via Decreasing ROS Production and Sustaining Mitochondrial Homeostasis

**Figure 3:**
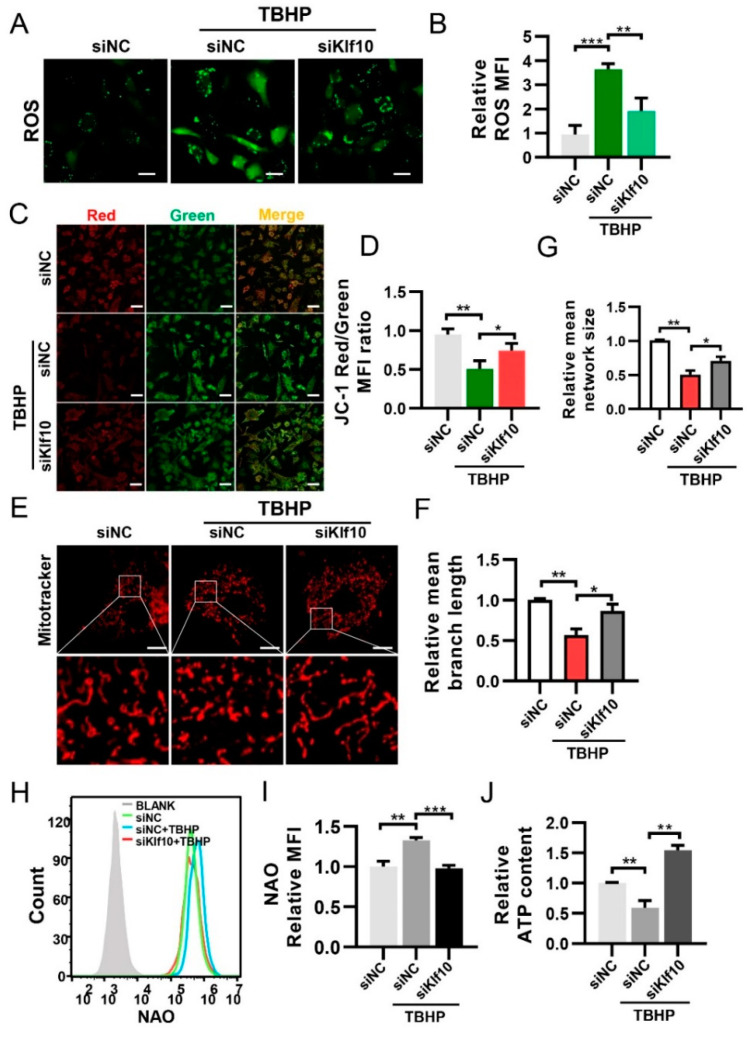
Downregulating Klf10 attenuated ROS production and sustained mitochondrial hemostasis. The murine chondrocytes were treated with TBHP with or without transfection with siKlf10. (**A**,**B**) Intracellular ROS of each group was determined by DCF fluorescence and the mean fluorescence intensity (MFI) was used for quantitative analysis (*n* = 3). Scale bar: 20 μm. (**C**,**D**) Mitochondrial membrane potential of each group was detected by JC-1. Red fluorescence indicated high mitochondrial membrane potential, whereas green fluorescence of JC-1 monomers indicated low mitochondrial membrane potential. The mitochondrial membrane potential was quantified by the ratio of red/green MFI (*n* = 3). Scale bar: 100 μm. (**E**–**G**) To visualize the morphology of mitochondria, the chondrocytes were stained with 100 nM MitoTracker Red for 30 min and observed using confocal laser scanning microscopy. The mean network size and mean branch length of mitochondria in each group were quantitatively analyzed using Fiji software (*n* = 3). Scale bar: 10 μm. (**H**,**I**) The chondrocytes were stained with NAO and the fluorescence was detected by flow cytometry. The mitochondrial mass was quantized by the MFI (*n* = 3). (**J**) Intracellular ATP content of each group was measured with the luminescence-based ATP assay kit (*n* = 3). Data are presented as mean  ±  SD. One-way ANOVA with Tukey’s post hoc test was used for comparisons among multiple groups. * *p* < 0.05, ** *p* < 0.01, *** *p* < 0.001.

ROS is known to be an important factor in cellular senescence. We found that downregulating Klf10 reduced ROS levels in TBHP-induced senescent cells (Figure 3A,B). The maintenance of intracellular ROS levels is primarily dependent on the balance between ROS production and its clearance by the antioxidant system [32]. Therefore, we tested the expression of superoxide dismutase 2 (Sod2) and catalase (Cat), but no significant difference was found when Klf10 was knocked down (Figure S1). For further investigation, the JC-1 probe was employed to assess mitochondrial membrane potential. Results of the ratio of red/green fluorescence intensity suggested that Δψm decreased in the TBHP group and was restored when Klf10 was knocked down (Figure 3C,D). Using MitoTracker as a mitochondrial fluorescent probe, mitochondrial fragments were found in senescent chondrocytes. However, when Klf10 was knocked down, the frequency of mitochondrial fragments was reduced (Figure 3E–G). Furthermore, mitochondrial mass was increased in senescent chondrocytes and was decreased by the downregulation of Klf10 (Figure 3H,I). Intracellular ATP content was reduced in senescent chondrocytes and was increased when Klf10 was knocked down (Figure 3J). These findings suggest that the dysfunctional mitochondria that accumulated in senescent chondrocytes were responsible for increased cellular ROS levels, and downregulation of Klf10 inhibited chondrocyte senescence by restoring mitochondrial function and reducing ROS generation.

### 2.4. Downregulating Klf10 Restored Autophagy Flux Suppressed in TBHP-Induced Senescent Chondrocytes

**Figure 4:**
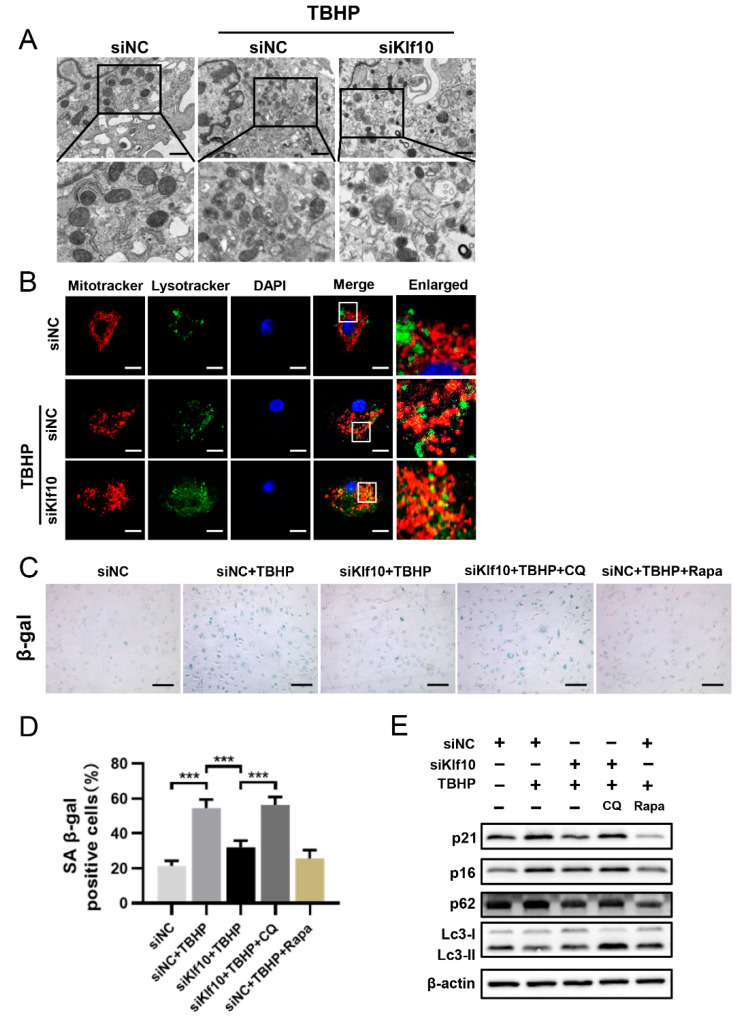
Downregulating Klf10 restored autophagy flux suppressed in TBHP-induced senescent chondrocytes. (**A**) The murine chondrocytes were treated with TBHP with or without transfection with siKlf10. The mitochondrial morphology of each group was analyzed by transmission electron microscope (TEM). The red arrow represents impaired mitochondria and the yellow triangle represents autophagolysosome. Scale bar: 1 μm. (**B**) After being treated with TBHP with or without transfection with siKlf10, the chondrocytes were stained with MitoTracker Red (100 nM, 30 min) and LysoTracker Green (75 nM, 30 min) to label the mitochondria and lysosomes. The co-localization of mitochondria and lysosomes was detected by laser scanning confocal microscopy. Scale bar: 20 μm. (**C**,**D**) The chondrocytes were pretreated with CQ or Rapa, followed by treatment with TBHP with or without transfection with siKlf10. SA-β-Gal staining was applied to detect senescent chondrocytes of different treatment groups (*n* = 3). Scale bar: 200 μm. (**E**) Protein expressions of p16, p21, Lc3, and p62 in different treatment groups were determined by Western blotting. Data are presented as the mean  ±  SD. One-way ANOVA with Tukey’s post hoc test was used for comparisons among multiple groups. *** *p* < 0.001.

To investigate the role of Klf10 in restoring dysfunctional mitochondria, TEM was applied to study the of mitochondrial morphology. As shown in Figure 4A, the mitochondria were normal and tubular in the control group but became swollen and impaired when treated with TBHP. Interestingly, autophagolysosome formation in the cytoplasm was observed when Klf10 was knocked down, suggesting the promotion of mitophagy. The activation of mitophagy was then confirmed by the co-localization of labeled mitochondria and lysosomes, which showed that knocking down Klf10 in TBHP-induced senescent chondrocytes promoted mitochondria and lysosome co-localization (Figure 4B). To explore the relationship between mitophagy and Klf10 and the role of mitophagy in chondrocyte senescence, CQ and Rapa were applied to block and activate autophagy. Results of SA-β-gal staining showed that knocking down Klf10 significantly inhibited TBHP-induced chondrocyte senescence, which was similar to the effect of rapamycin (Figure 4C,D). When the mitophagy induced by knocking down Klf10 was blocked by CQ, the positive rate of SA-β-gal staining increased significantly, demonstrating that the inhibitory effect of Klf10 knockdown on chondrocyte senescence could be eliminated when mitophagy was blocked (Figure 4C,D). The results of Western blot analysis showed that when autophagy was inhibited, the expression levels of p16 and p21 increased, and when autophagy was activated, p16 and p21 expression was significantly reduced (Figure 4E). Taken together, these results suggest that mitophagy was suppressed in TBHP-induced senescence, and the downregulation of Klf10 could attenuate senescence by promoting mitophagy.

### 2.5. Downregulation of Klf10 Promoted Mitophagy by Upregulating Bnip3

**Figure 5:**
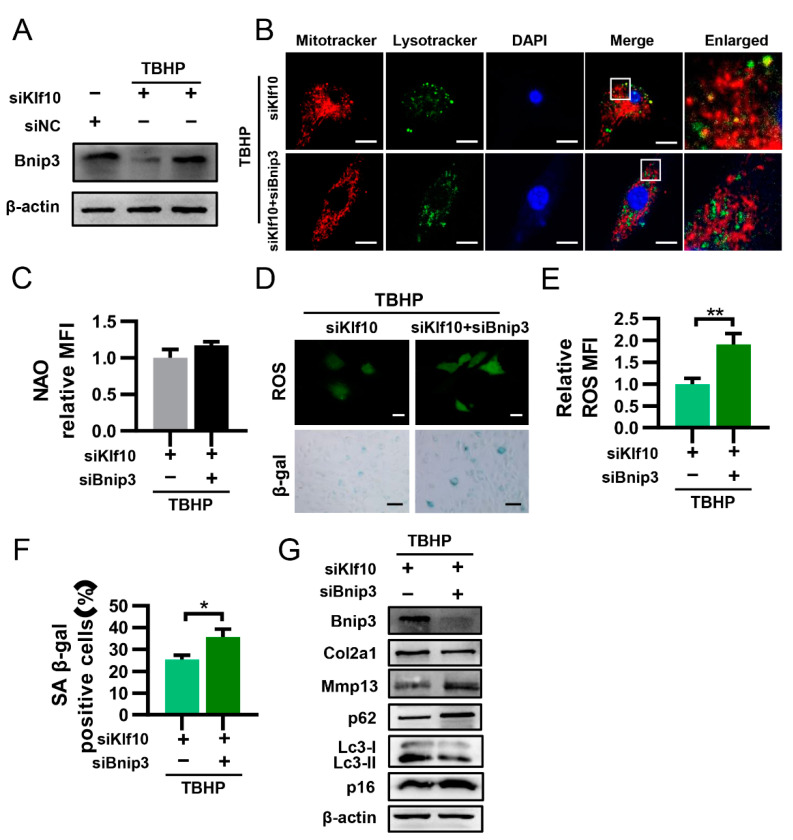
Downregulation of Klf10 promoted mitophagy by upregulating Bnip3. (**A**) After treating murine chondrocytes with TBHP with or without transfection with siKlf10, the protein expression of Bnip3 in each group was detected by Western blotting (*n* = 3). (**B**) The chondrocytes were treated with TBHP and transfected with siKlf10 or both siKlf10 and siBnip3, followed by staining with MitoTracker Red (100 nM, 30 min) and LysoTracker Green (75 nM, 30 min) to label mitochondria and lysosomes. The co-localization of mitochondria and lysosomes was detected by confocal laser scanning microscopy. Scale bar: 20 μm. (**C**) The chondrocytes were stained with NAO and detected by flow cytometry. The mitochondrial mass was quantized by the MFI (*n* = 3). (**D**–**F**) Representative images of intracellular ROS and senescent chondrocytes of both groups. The intracellular ROS was quantized by the MFI of DCF and senescent chondrocytes were quantized by SA-β-Gal positive cells (*n* = 3). Scale bar: 20 μm. (**G**) Protein expression of Col2a1, Mmp13, p16, p21, Lc3, p62, and Bnip3 in both groups was determined by Western blotting. Data are presented as the mean  ±  SD. Student’s *t* test was used for comparisons between two groups. * *p* < 0.05, ** *p* < 0.01.

The BCL2-interacting protein 3 (BNIP3) is a Bcl-2 family protein that primarily localizes at the mitochondrial outer membrane, which serves as a mitophagy receptor that interacts with LC3 on the autophagosome and targets dysfunctional mitochondria [33]. In our study, Western blot analysis showed that the expression of Bnip3 protein was significantly reduced in TBHP-induced senescent chondrocytes and was restored by knocking down Klf10 (Figure 5A). To further verify that downregulation of Klf10 promoted mitophagy through Bnip3, we applied Bnip3 siRNA. The fluorescence results indicated that co-knockdown of Klf10 and Bnip3 significantly reduced the co-localization of mitochondria and lysosomes (Figure 5B). The mitochondrial mass exhibited a slight but insignificant increase (Figure 5C). Furthermore, co-knockdown of Klf10 and Bnip3 significantly increased intracellular ROS levels and the positive rate of senescent chondrocytes (Figure 5D–F). In addition, Western blotting results showed that after co-knockdown of Klf10 and Bnip3, Col2a1 and Lc3II/I were decreased while the expression of Mmp13, p62, p16, and p21 increased, indicating that autophagy flux was inhibited and senescence was promoted (Figure 5G). Taken together, these results suggest that knocking down Klf10 activated mitophagy by promoting the expression of Bnip3.

## 3. Materials and Methods

### 3.1. Isolation and Culture of Murine Chondrocytes

The murine chondrocytes were isolated and cultured according to the previously reported method [34]. Briefly, cartilage from the knee joints of five-day-old newborn C57BL/6 mice was collected following humane euthanasia. Then, the cartilage was divided into pieces and incubated in collagenase D solution (3 mg/mL) for 45 min at 37 °C and 5% CO_2_, followed by retrieval of cartilage pieces and their digestion overnight at 37 °C in collagenase D solution (0.5 mg/mL). The chondrocytes were then cultured in DMEM/F12 medium supplemented with 10% fetal bovine serum (FBS). The cells were passaged or seeded for further experiments when they reached 80% confluence.

### 3.2. Chondrocyte Treatment and siRNA Transfection

Tert-butyl hydroperoxide (TBHP) is an initiator of free radical reactions and has been applied to induce premature senescence in multiple cells [35]. To explore the dose of TBHP (Sigma-Aldrich, St. Louis, MI, USA) required to induce chondrocyte senescence, murine chondrocytes were exposed to 0, 25, or 50 μM TBHP for 48 h. To study the relationship between autophagy flux and senescence, chondrocytes were pretreated with autophagy inhibitor chloroquine (CQ, Sigma-Aldrich, St. Louis, MI, USA) and autophagy activator rapamycin (Rapa, MedChemExpress, Monmouth Junction, NJ, USA). The siRNAs for Klf10 and Bnip3 were designed and constructed by GenePharma (Shanghai, China). The manufacturer’s instructions were followed in the use of siRNA-Mate for siRNA transfection, and the sequences of Klf10-siRNA, Bnip3-siRNA, and the negative control are listed in Table S1.

### 3.3. Western Blot Analysis

The cells were lysed by RIPA (Solarbio, Beijing, China) with 1 mM PMSF (Solarbio, Beijing, China). Protein-extract supernatants were quantified using a BCA protein assay kit. A total of 20 μg protein was subjected to electrophoresis using SDS-PAGE and transferred to PVDF (Bio-Rad, Hercules, CA, USA). After blocking in 5% non-fat milk for 1 h at room temperature, membranes were incubated overnight at 4 °C with primary antibodies against Col2a1 (ABclonal, Wuhan, China, 1:1000), Mmp13 (Proteintech Group, Rosemont, IL, USA, 1:1000), p16 (Abcam, Cambridge, UK, 1:1000), p21 (ABclonal, Wuhan, China, 1:1000), p62 (ABclonal, Wuhan, China, 1:1000), Lc3 (Cell Signaling Technology, Danvers, MA, USA, 1:1000), Klf10 (Santa Cruz Biotechnology, Paso Robles, CA, USA, 1:1000), Bnip3 (Santa Cruz Biotechnology, Paso Robles, CA, USA, 1:1000), and β-actin (Proteintech Group, Rosemont, IL, USA, 1:5000), followed by incubation of the blots in HRP-conjugated secondary antibodies. The representative images were acquired by ECL reagents (Solarbio, Beijing, China) using an iBright 1500 imaging system (Thermo Fisher, Waltham, MA, USA).

### 3.4. RNA Extraction and Quantitative Real-Time PCR (RT-qPCR)

RNA was extracted using a total RNA isolation kit (Omega Bio-Tek, Norcross, GA, USA) and reversely transcribed to cDNA using the RevertAid First Strand cDNA synthesis kit (Thermo Fisher, Waltham, MA, USA) following the manufacturer’s instructions. Quantitative PCR was performed using Forget-MeNot™ EvaGreen^®^ qPCR master mix (Biotium, Fremont, CA, USA) using a CFX96™ real-time PCR detection system (Bio-Rad, Hercules, CA, USA). Expression levels of the target genes were calculated using the 2^−ΔΔCT^ method. The sequences of the primers are listed in Table S2.

### 3.5. SA-β-Gal Staining

An SA-β-gal staining kit (Solarbio, Beijing, China) was utilized to detect chondrocyte senescence according to the manufacturer’s instructions. The images of SA-β-gal staining were taken under a microscope (Olympus, Tokyo, Japan), and the results quantified according to the frequency of positive cells.

### 3.6. EdU (5-Ethynyl-2′-deoxyuridine) Assay

An EdU cell proliferation kit with Alexa Fluor 555 (Beyotime Biotechnology, Shanghai, China) was used for EdU staining to detect chondrocyte proliferation, following the manufacturer’s instructions. Images were captured using fluorescence microscopy (Olympus, Tokyo, Japan), and the results were quantified based on the percentage of EdU-positive cells.

### 3.7. Determination of Intracellular ATP

Intracellular ATP content was measured utilizing a luminescence-based ATP assay kit (Beyotime Biotechnology, Shanghai, China) according to the manufacturer’s instructions. The fluorescence intensity was measured using a multimode plate reader (PerkinElmer, Boston, MA, USA). ATP concentrations were determined using a standard curve and normalized by protein content.

### 3.8. Transmission Electron Microscopy (TEM)

The chondrocytes were trypsinized and fixed in 2.5% glutaraldehyde for 2 h at 4 °C, followed by treatment with 1% osmium tetroxide. They were then washed with PBS, dehydrated in a graded concentration of ethanol and acetone, and embedded in the Epon mixture. Then, the cells were sliced into ultrathin sections and respective sections were photographed using a transmission electron microscope (Hitachi, Tokyo, Japan).

### 3.9. Immunofluorescence

The chondrocytes were seeded in dishes and fixed with 4% paraformaldehyde before treatment with 0.3% Triton X-100 for 15 min. After blocking with 10% goat serum for 1 h at 37 °C, the cells were incubated with the primary antibody overnight at 4 °C. The cells were then incubated for 1 h at room temperature with fluorescein-labeled secondary antibodies (Alexa Fluor 488 or Alexa Fluor 594) in the dark, before being labeled with DAPI (4′,6-diamidino-2-phenylindole) for 5 min. The fluorescent images were captured using a confocal laser scanning microscope (Zeiss, Oberkochen, Germany) and the fluorescence intensity was measured for quantitative analysis.

### 3.10. Assessment of Mitochondria and Lysosome Co-Localization

MitoTracker Red (Thermo Fisher, Waltham, MA, USA) and LysoTracker Green (Beyotime Biotechnology, Shanghai, China) were applied to label mitochondria and lysosomes in live chondrocytes, according to the manufacturers’ instructions. The images were photographed using a confocal laser scanning microscope (Zeiss, Oberkochen, Germany).

### 3.11. Measurement of Cellular ROS

The cellular ROS level was determined by incubating cells with CM-H_2_DCF-DA (Thermo Fisher, Waltham, MA, USA) for 20 min at 37 °C. The photographs were taken using a confocal laser scanning microscope (Zeiss, Oberkochen, Germany) and the fluorescence intensity was measured for quantitative analysis.

### 3.12. Mitochondrial Mass Analysis

10-N-nonyl-acridine orange (NAO) is a fluorescent probe commonly employed to assess mitochondrial mass [36]. For this purpose, cells were stained with NAO (Thermo Fisher, Waltham, MA, USA) for 20 min at 37 °C, washed twice with PBS, and analyzed using flow cytometry (Beckman, Brea, CA, USA) at an excitation wavelength of 495 nm and an emission wavelength of 519 nm.

### 3.13. Mitochondrial Membrane Potential (Δψm) Detection

The mitochondrial Δψm of chondrocytes was detected using the fluorescent probe JC-1 (Beyotime Biotechnology, Shanghai, China), according to the manufacturer’s instructions. Chondrocytes were incubated with JC-1 for 30 min at 37 °C in the dark, followed by washing twice with PBS. The fluorescence was visualized using a confocal laser scanning microscope (Zeiss, Oberkochen, Germany) at 488 nm for green fluorescence and 525 nm for red fluorescence. 

### 3.14. Human Samples Collection and Construction of Mouse DMM Model of OA

The OA cartilage was collected from OA patients undergoing total hip arthroplasty, and the cartilage of the control group was collected from patients undergoing total hip arthroplasty due to femoral neck fracture.

For the mouse DMM model of OA, the mice were anesthetized with pentobarbital sodium intraperitoneally, followed by longitudinal incision on the inner side of the patellar ligament of the knee joint of the mouse’s right hind limb, and the patellar ligament was pulled outward with silk thread for blunt separation of the subpatellar tissue and to expose the joint space. The medial meniscotibial ligament (MMTL) was identified and removed using a microsurgical blade. After opening the joint cavity, the MMTL in the control group was not removed, while rest of the procedure was same as for the DMM group. The animals were sacrificed eight weeks after DMM surgery, and their joints were collected for the subsequent experiments.

### 3.15. Statistical Analysis

The data are presented as mean ± standard deviation (SD). Statistical significance was evaluated using Student’s *t* test between two groups or one-way analysis of variance (ANOVA) with Tukey’s post hoc test among multiple groups. *p* < 0.05 was considered statistically significant.

## 4. Discussion

OA is the most common degenerative joint disease in aging people, primarily triggered by external force caused trauma or age-related cartilage damage [2,37]. Obesity, genetics, and aging are common risk factors for OA. Cellular senescence is involved in multiple age-related diseases, and is associated with elevated intracellular ROS, accumulated dysfunctional mitochondria, and increased mitochondrial mass [23,38]. A previous study reported that transplanting senescent cells into the knee joint can induce an OA-like state in mice [39]. In contrast, clearance of senescent cells attenuated the development of post-traumatic OA [12]. Therefore, it is necessary to identifying the mechanism that underlies senescence, because suppressing the process of senescence may be a potential therapeutic method for OA. In the current study, we established senescent chondrocytes using TBHP and identified the upregulation of Klf10 in these senescent chondrocytes. The knockdown of Klf10 inhibited the expression of p16, p21, and degeneration of the extracellular matrix (ECM).

Mitochondrial homeostasis plays an important role in cellular senescence. Cumulative evidence has suggested increased mitochondrial mass in senescent cells [22,38]. However, mitochondria are less functional in senescent cells, despite being more abundant [40]. Dysfunctional mitochondria, characterized by disrupted membrane potential, increased production of superoxide anions and hydrogen peroxide, and decreased ATP production, have been identified as the primary cause of senescence and SASP generation [20,40,41]. The electron transport chain located in the inner membrane of the mitochondria is the principal site of ATP production via oxidative phosphorylation, and is the primary site for ROS generation [42,43]. In normal cells, the content of ROS is strictly regulated. However, under some abnormal conditions, excessive intracellular ROS are generated, followed by oxidative damage to DNA and proteins, facilitating the process of senescence. Previous studies have shown the multiple roles of Klf10 in regulating mitochondrial homeostasis [44,45,46,47]. According to Jin et al., Klf10 overexpression can cause disruption of mitochondrial membrane potential and the release of cytochrome c from mitochondria into the cytosol [42]. Similarly, Ribeiro et al. reported that upregulating Klf10 caused an increase in ROS production and a loss of mitochondrial membrane potential, which resulted in cell apoptosis [45]. In a recent study, Kammoun et al. found that Klf10 KO mice exhibited altered mitochondrial function, including decreased complex I, cytochrome c oxidase (COX), and citrate synthase (CS) activity, as well as a significant decrease in the mitochondrial number [46]. In this study, we revealed that knocking down Klf10 in senescent chondrocytes effectively restored the disrupted mitochondrial membrane potential and maintained mitochondrial mass, accompanied by a simultaneous reduction of ROS generation. Consistent with previous studies, our data provide evidence that Klf10 is involved in maintaining mitochondrial homeostasis.

Mitophagy, the selective degradation of mitochondria, is primarily responsible for the clearance of dysfunctional mitochondria [48], contributing to mitochondrial turnover and quantity control in various cell types [49,50]. Recent evidence suggests that impaired mitophagy led to cell senescence, while restoration of the mitophagy flux effectively inhibited cell senescence [24,25,51]. Araya et al. showed that Prkn KO mice exhibited accumulations of damaged mitochondria and accelerated senescence in airway epithelial cells; overexpression of Prkn by pirfenidone was sufficient to activate mitophagy, causing the consequent reduction of mitochondrial ROS production and attenuating cellular senescence [24]. Similarly, in OA chondrocytes, Parkin could mediate the elimination of damaged mitochondria and reduce ROS levels by activating autophagy [52]. Klf10 has been implicated in autophagy modulation by regulating the interaction between Beclin and PI3KC3 for autophagosome formation [31]. Furthermore, KLF10 has been linked to the regulation of autophagy-related genes including heme oxygenase-1 (Ho-1) and proliferator-activated receptor gamma coactivator-1 alpha (Pgc-1alpha) [30]. A relationship between Klf10, senescence, and autophagy can thereby be hypothesized. We examined autophagy flux in Klf10 knockdown chondrocytes, and the results showed that downregulation of Klf10 could restore compromised mitophagy flux to eliminate dysfunctional mitochondria in senescent chondrocytes. BNIP3 is known to be a receptor of mitophagy [33]. Tang et al. found that reduced Bnip3 expression led to suppressed mitophagy, accumulation of damaged mitochondria, and increased ROS production in mouse proximal tubule cells, while BNIP3 overexpression could reverse compromised mitophagy and restore mitochondrial homeostasis [53]. In line with previous findings, our study discovered that co-knockdown of Bnip3 and Klf10 suppressed the mitophagy restored by Klf10 in senescent chondrocytes, implying that Klf10 regulated mitophagy via Bnip3.

However, there are several limitations to the current study. First, although our results provide evidence that siRNA-mediated Klf10 knockdown could attenuate chondrocyte senescence, more solid evidence from Klf10 KO mice is required. Second, most of our results were collected from experiments with murine chondrocytes, and further research in human chondrocytes remains necessary. Third, the mechanism by which Klf10 regulates Bnip3 in senescent chondrocytes remains unclear. Given the important role of Klf10 in regulating chondrocyte senescence and maintaining chondrocyte homeostasis, further studies are required to investigate how Klf10 activates Bnip3-mediated mitophagy to eliminate dysfunctional mitochondria.

## 5. Conclusions

In conclusion, our findings show that Klf10 expression was upregulated in senescent chondrocytes and OA cartilage. Downregulation of Klf10 attenuated TBHP-induced senescence, reduced cellular ROS levels, and restored mitochondrial homeostasis by modulating mitophagy. These findings suggest that Klf10 plays a functional role in chondrocyte senescence and may be a potential therapeutic target for OA.

## Data Availability

The data are contained within the article.

## Supplementary Materials

The following supporting information can be downloaded at: https://www.mdpi.com/article/10.3390/molecules28030924/s1, Table S1: siRNA sequences; Table S2: Primer sequences; Figure S1: RT-qPCR results.

## References

[1] Cui A., Li H., Wang D., Zhong J., Chen Y., Lu H. (2020). Global, regional prevalence, incidence and risk factors of knee osteoarthritis in population-based studies. EClinicalMedicine.

[2] Vina E.R., Kwoh C.K. (2018). Epidemiology of osteoarthritis: Literature update. Curr. Opin. Rheumatol..

[3] Rhinn M., Ritschka B., Keyes W.M. (2019). Cellular senescence in development, regeneration and disease. Development.

[4] Hernandez-Segura A., Nehme J., Demaria M. (2018). Hallmarks of Cellular Senescence. Trends Cell Biol..

[5] Salama R., Sadaie M., Hoare M., Narita M. (2014). Cellular senescence and its effector programs. Genes Dev..

[6] Birch J., Gil J. (2020). Senescence and the SASP: Many therapeutic avenues. Genes Dev..

[7] Tchkonia T., Zhu Y., van Deursen J., Campisi J., Kirkland J.L. (2013). Cellular senescence and the senescent secretory phenotype: Therapeutic opportunities. J. Clin. Investig..

[8] Faust H.J., Zhang H., Han J., Wolf M.T., Jeon O.H., Sadtler K., Peña A.N., Chung L., Maestas D.R., Tam A.J. (2020). IL-17 and immunologically induced senescence regulate response to injury in osteoarthritis. J. Clin. Investig..

[9] Loeser R.F., Kelley K.L., Armstrong A., Collins J.A., Diekman B.O., Carlson C.S. (2020). Deletion of JNK Enhances Senescence in Joint Tissues and Increases the Severity of Age-Related Osteoarthritis in Mice. Arthritis Rheumatol..

[10] Martin J.A., Buckwalter J.A. (2003). The role of chondrocyte senescence in the pathogenesis of osteoarthritis and in limiting cartilage repair. J. Bone Jt. Surg. Am. Vol..

[11] Gao S.G., Zeng C., Li L.J., Luo W., Zhang F.J., Tian J., Cheng C., Tu M., Xiong Y.L., Jiang W. (2016). Correlation between senescence-associated beta-galactosidase expression in articular cartilage and disease severity of patients with knee osteoarthritis. Int. J. Rheum. Dis..

[12] Jeon O.H., Kim C., Laberge R.M., Demaria M., Rathod S., Vasserot A.P., Chung J.W., Kim D.H., Poon Y., David N. (2017). Local clearance of senescent cells attenuates the development of post-traumatic osteoarthritis and creates a pro-regenerative environment. Nat. Med..

[13] Guo Z., Wang G., Wu B., Chou W.C., Cheng L., Zhou C., Lou J., Wu D., Su L., Zheng J. (2020). DCAF1 regulates Treg senescence via the ROS axis during immunological aging. J. Clin. Investig..

[14] Niu F., Cui X., Zhao P., Sun M., Yang B., Deyholos M.K., Li Y., Zhao X., Jiang Y.Q. (2020). WRKY42 transcription factor positively regulates leaf senescence through modulating SA and ROS synthesis in Arabidopsis thaliana. Plant J. Cell Mol. Biol..

[15] Parzych K.R., Klionsky D.J. (2014). An overview of autophagy: Morphology, mechanism, and regulation. Antioxid. Redox Signal..

[16] García-Prat L., Martínez-Vicente M., Perdiguero E., Ortet L., Rodríguez-Ubreva J., Rebollo E., Ruiz-Bonilla V., Gutarra S., Ballestar E., Serrano A.L. (2016). Autophagy maintains stemness by preventing senescence. Nature.

[17] Zhang H., Simon A.K. (2020). Polyamines reverse immune senescence via the translational control of autophagy. Autophagy.

[18] Moreno-Blas D., Gorostieta-Salas E., Pommer-Alba A., Muciño-Hernández G., Gerónimo-Olvera C., Maciel-Barón L.A., Konigsberg M., Massieu L., Castro-Obregón S. (2019). Cortical neurons develop a senescence-like phenotype promoted by dysfunctional autophagy. Aging.

[19] Daskalaki I., Tavernarakis N. (2020). Mitochondrial biogenesis in organismal senescence and neurodegeneration. Mech. Ageing Dev..

[20] Janikiewicz J., Szymański J., Malinska D., Patalas-Krawczyk P., Michalska B., Duszyński J., Giorgi C., Bonora M., Dobrzyn A., Wieckowski M.R. (2018). Mitochondria-associated membranes in aging and senescence: Structure, function, and dynamics. Cell Death Dis..

[21] Chapman J., Fielder E., Passos J.F. (2019). Mitochondrial dysfunction and cell senescence: Deciphering a complex relationship. FEBS Lett..

[22] Callender L.A., Carroll E.C., Bober E.A., Akbar A.N., Solito E., Henson S.M. (2020). Mitochondrial mass governs the extent of human T cell senescence. Aging Cell.

[23] Moiseeva O., Bourdeau V., Roux A., Deschênes-Simard X., Ferbeyre G. (2009). Mitochondrial dysfunction contributes to oncogene-induced senescence. Mol. Cell. Biol..

[24] Araya J., Tsubouchi K., Sato N., Ito S., Minagawa S., Hara H., Hosaka Y., Ichikawa A., Saito N., Kadota T. (2019). PRKN-regulated mitophagy and cellular senescence during COPD pathogenesis. Autophagy.

[25] Chen K., Dai H., Yuan J., Chen J., Lin L., Zhang W., Wang L., Zhang J., Li K., He Y. (2018). Optineurin-mediated mitophagy protects renal tubular epithelial cells against accelerated senescence in diabetic nephropathy. Cell Death Dis..

[26] Subramaniam M., Harris S.A., Oursler M.J., Rasmussen K., Riggs B.L., Spelsberg T.C. (1995). Identification of a novel TGF-beta-regulated gene encoding a putative zinc finger protein in human osteoblasts. Nucleic Acids Res..

[27] Parakati R., DiMario J.X. (2013). Repression of myoblast proliferation and fibroblast growth factor receptor 1 promoter activity by KLF10 protein. J. Biol. Chem..

[28] Hsu C.F., Sui C.L., Wu W.C., Wang J.J., Yang D.H., Chen Y.C., Yu W.C., Chang H.S. (2011). Klf10 induces cell apoptosis through modulation of BI-1 expression and Ca2+ homeostasis in estrogen-responding adenocarcinoma cells. Int. J. Biochem. Cell Biol..

[29] Chen Z., Li W., Wang H., Wan C., Luo D., Deng S., Chen H., Chen S. (2016). Klf10 regulates odontoblast differentiation and mineralization via promoting expression of dentin matrix protein 1 and dentin sialophosphoprotein genes. Cell Tissue Res..

[30] Zhou J., Zhang L., Zheng B., Zhang L., Qin Y., Zhang X., Yang Z., Nie Z., Yang G., Yu J. (2020). Salvia miltiorrhiza bunge exerts anti-oxidative effects through inhibiting KLF10 expression in vascular smooth muscle cells exposed to high glucose. J. Ethnopharmacol..

[31] Chang V.H., Tsai Y.C., Tsai Y.L., Peng S.L., Chen S.L., Chang T.M., Yu W.C., Ch’ang H.J. (2017). Krüpple-like factor 10 regulates radio-sensitivity of pancreatic cancer via UV radiation resistance-associated gene. Radiother. Oncol. J. Eur. Soc. Ther. Radiol. Oncol..

[32] Zhang L., Wang X., Cueto R., Effi C., Zhang Y., Tan H., Qin X., Ji Y., Yang X., Wang H. (2019). Biochemical basis and metabolic interplay of redox regulation. Redox Biol..

[33] O’Sullivan T.E., Johnson L.R., Kang H.H., Sun J.C. (2015). BNIP3- and BNIP3L-Mediated Mitophagy Promotes the Generation of Natural Killer Cell Memory. Immunity.

[34] Gosset M., Berenbaum F., Thirion S., Jacques C. (2008). Primary culture and phenotyping of murine chondrocytes. Nat. Protoc..

[35] Wedel S., Martic I., Hrapovic N., Fabre S., Madreiter-Sokolowski C.T., Haller T., Pierer G., Ploner C., Jansen-Dürr P., Cavinato M. (2020). tBHP treatment as a model for cellular senescence and pollution-induced skin aging. Mech. Ageing Dev..

[36] Doherty E., Perl A. (2017). Measurement of Mitochondrial Mass by Flow Cytometry during Oxidative Stress. React. Oxyg. Species.

[37] Rim Y.A., Nam Y., Ju J.H. (2020). The Role of Chondrocyte Hypertrophy and Senescence in Osteoarthritis Initiation and Progression. Int. J. Mol. Sci..

[38] Korolchuk V.I., Miwa S., Carroll B., von Zglinicki T. (2017). Mitochondria in Cell Senescence: Is Mitophagy the Weakest Link?. EBioMedicine.

[39] Xu M., Bradley E.W., Weivoda M.M., Hwang S.M., Pirtskhalava T., Decklever T., Curran G.L., Ogrodnik M., Jurk D., Johnson K.O. (2017). Transplanted Senescent Cells Induce an Osteoarthritis-Like Condition in Mice. J. Gerontol. Ser. A Biol. Sci. Med. Sci..

[40] Gorgoulis V., Adams P.D., Alimonti A., Bennett D.C., Bischof O., Bishop C., Campisi J., Collado M., Evangelou K., Ferbeyre G. (2019). Cellular Senescence: Defining a Path Forward. Cell.

[41] Kaplon J., Zheng L., Meissl K., Chaneton B., Selivanov V.A., Mackay G., van der Burg S.H., Verdegaal E.M., Cascante M., Shlomi T. (2013). A key role for mitochondrial gatekeeper pyruvate dehydrogenase in oncogene-induced senescence. Nature.

[42] Brookes P.S., Yoon Y., Robotham J.L., Anders M.W., Sheu S.S. (2004). Calcium, ATP, and ROS: A mitochondrial love-hate triangle. Am. J. Physiology. Cell Physiol..

[43] Li X., Fang P., Yang W.Y., Chan K., Lavallee M., Xu K., Gao T., Wang H., Yang X. (2017). Mitochondrial ROS, uncoupled from ATP synthesis, determine endothelial activation for both physiological recruitment of patrolling cells and pathological recruitment of inflammatory cells. Can. J. Physiol. Pharmacol..

[44] Jin W., Di G., Li J., Chen Y., Li W., Wu J., Cheng T., Yao M., Shao Z. (2007). TIEG1 induces apoptosis through mitochondrial apoptotic pathway and promotes apoptosis induced by homoharringtonine and velcade. FEBS Lett..

[45] Ribeiro A., Bronk S.F., Roberts P.J., Urrutia R., Gores G.J. (1999). The transforming growth factor beta(1)-inducible transcription factor TIEG1, mediates apoptosis through oxidative stress. Hepatology.

[46] Kammoun M., Piquereau J., Nadal-Desbarats L., Même S., Beuvin M., Bonne G., Veksler V., Le Fur Y., Pouletaut P., Même W. (2020). Novel role of Tieg1 in muscle metabolism and mitochondrial oxidative capacities. Acta Physiol..

[47] Wara A.K., Wang S., Wu C., Fang F., Haemmig S., Weber B.N., Aydogan C.O., Tesmenitsky Y., Aliakbarian H., Hawse J.R. (2020). KLF10 Deficiency in CD4(+) T Cells Triggers Obesity, Insulin Resistance, and Fatty Liver. Cell Rep..

[48] Dombi E., Mortiboys H., Poulton J. (2018). Modulating Mitophagy in Mitochondrial Disease. Curr. Med. Chem..

[49] Onishi M., Yamano K., Sato M., Matsuda N., Okamoto K. (2021). Molecular mechanisms and physiological functions of mitophagy. EMBO J..

[50] Ma K., Chen G., Li W., Kepp O., Zhu Y., Chen Q. (2020). Mitophagy, Mitochondrial Homeostasis, and Cell Fate. Front. Cell Dev. Biol..

[51] Hu S., Zhang C., Ni L., Huang C., Chen D., Shi K., Jin H., Zhang K., Li Y., Xie L. (2020). Stabilization of HIF-1α alleviates osteoarthritis via enhancing mitophagy. Cell Death Dis..

[52] Ansari M.Y., Khan N.M., Ahmad I., Haqqi T.M. (2018). Parkin clearance of dysfunctional mitochondria regulates ROS levels and increases survival of human chondrocytes. Osteoarthr. Cartil..

[53] Tang C., Han H., Liu Z., Liu Y., Yin L., Cai J., He L., Liu Y., Chen G., Zhang Z. (2019). Activation of BNIP3-mediated mitophagy protects against renal ischemia-reperfusion injury. Cell Death Dis..

