# Supplementary material for: Inhibition of Klf10 Attenuates Oxidative Stress-Induced Senescence of Chondrocytes via Modulating Mitophagy
Source: Molecules. 2023 Jan 17;28(3):924. doi: 10.3390/molecules28030924 (PMC9921806; doi:10.3390/molecules28030924)

**Table S1. siRNA sequences**

| Genes | Sense (5′-3′) |
|---|---|
| *Klf10* #1 | GCUUCUCUCCAGCAAGCUUTT |
| *Klf10* #2 | GGGUCAAAUCUGACUGCAUTT |
| *Klf10* #3 | GCCACCUAUCAGCCAAGAATT |
| *Bnip3* | CAGCCUCCGUCUCUAUUUATTT |
| Negative control | UUCUCCGAACGUGUCACGUTT |

**Table S2. Primer sequences**

| Genes | Forward (5'-3') | Reverse (5'-3') |
|---|---|---|
| *Klf10* | ATGCTCAACTTCGGCGCTT | CGCTTCCACCGCTTCAAAG |
| *Sod2* | CAGACCTGCCTTACGACTATGG | GCAGGATGGTAGTATGATT |
| *Cat* | AGCGACCAGATGAAGCAGTG | TCCGCTCTCTGTCAAAGTGTG |
| *Il-6* | TAGTCCTTCCTACCCCAATTTCC | TTGGTCCTTAGCCACTCCTTC |
| *Cxcl10* | CCAAGTGCTGCCGTCATTTTC | GGCTCGCAGGGATGATTTCAA |
| *Mcp1* | TTAAAAACCTGGATCGGAACCAA | GCATTAGCTTCAGATTTACGGGT |
| *Mmp3* | ACATGGAGACTTTGTCCCTTTTG | TTGGCTGAGTGGTAGAGTCCC |
| *β-actin* | CTCTGGCTCCTAGCACCATGAAGA | GTAAAACGCAGCTCAGTAACAGTCCG |

**Figure S1. RT-qPCR results.**

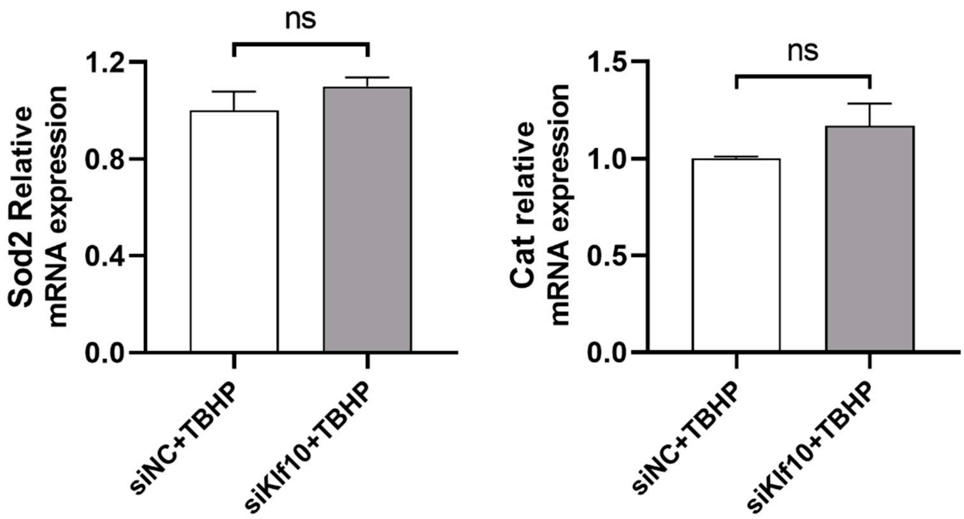

Supplement: Supplementary file 1 [file molecules-28-00924-s001.zip › molecules-2062457-supplementary.pdf]
